# A Highly Sensitive, Reliable, and High‐Temperature‐Resistant Flexible Pressure Sensor Based on Ceramic Nanofibers

**DOI:** 10.1002/advs.202000258

**Published:** 2020-07-27

**Authors:** Min Fu, Jianming Zhang, Yuming Jin, Yue Zhao, Siya Huang, Chuan Fei Guo

**Affiliations:** ^1^ SUSTech Academy for Advanced Interdisciplinary Studies Department of Materials Science and Engineering Department of Physics Southern University of Science and Technology Shenzhen Guangdong 518055 P. R. China; ^2^ Department of Materials Science and Engineering Southern University of Science and Technology Shenzhen Guangdong 518055 P. R. China; ^3^ Department of Physics Southern University of Science and Technology Shenzhen Guangdong 518055 P. R. China; ^4^ Institute for Advanced Study Shenzhen University Guangdong 518060 P. R. China

**Keywords:** ceramic nanofibers, flexible electronics, flexible pressure sensors, health monitoring, high‐temperature‐resistant devices

## Abstract

Flexible pressure sensors are essential components for soft electronics by providing physiological monitoring capability for wearables and tactile perceptions for soft robotics. Flexible pressure sensors with reliable performance are highly desired yet challenging to construct to meet the requirements of practical applications in daily activities and even harsh environments, such as high temperatures. This work describes a highly sensitive and reliable capacitive pressure sensor based on flexible ceramic nanofibrous networks with high structural elasticity, which minimizes performance degradation commonly seen in polymer‐based sensors because of the viscoelastic behavior of polymers. Such ceramic pressure sensors exhibit high sensitivity (≈4.4 kPa^−1^), ultralow limit of detection (<0.8 Pa), fast response speed (<16 ms) as well as low fatigue over 50 000 loading/unloading cycles. The high stability is attributed to the excellent mechanical stability of the ceramic nanofibrous network. By employing textile‐based electrodes, a fully breathable and wearable ceramic pressure sensor is demonstrated for real‐time health monitoring and motion detection. Owing to the high‐temperature resistance of ceramics, the ceramic nanofibrous network sensor can function properly at temperatures up to 370 °C, showing great promise for harsh environment applications.

## Graphical abstract

A highly sensitive and reliable pressure sensor based on flexible ceramic nanofibrous networks is designed in an extra lightweight, compliant, and breathable form, which is demonstrated to be suitable for continuous health monitoring and motion detection during long‐term wearing. Moreover, the sensor can function properly under high temperatures, showing great promise for applications under extreme conditions.

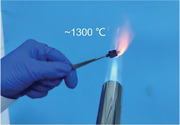

Driven by the increasing demand for bioinspired soft electronics,^[^ ^1^, ^2^, ^3^ ^]^ skin‐like flexible sensors are receiving unprecedented attention for their emerging applications in biomedical fields,^[^ ^4^, ^5^ ^]^ human–machine interaction,^[^ ^6^, ^7^ ^]^ soft robotics,^[^ ^8^, ^9^ ^]^ etc. By wearing flexible pressure sensors on different parts of a human body, a myriad of vital physiological signals (e.g., blood pressure, heart rate, and intraocular pressure) and body motions (e.g., eye blinking, walking, jumping, and squatting) can be tracked and analyzed in real‐time, offering a more effective and efficient way of accessing healthcare on a home basis.^[^ ^10^, ^11^ ^]^ Such a concept of decentralized healthcare delivery is expected to revolutionize the traditional way we receive healthcare services from a centralized hospital.

To ensure the accuracy of health/motion assessment during daily activities for long‐term use, consistent, and reliable performance is a prerequisite for wearable sensing devices.^[^ ^12^, ^13^ ^]^ Besides, since direct contact with bulky and airtight devices may cause discomfort and even inflammation to skins,^[^ ^14^ ^]^ flexible sensors that are lightweight^[^ ^15^ ^]^ and breathable,^[^ ^16^ ^]^ are expected for mobile and comfortable daily wearing. In particular, wearable sensors that can be used in harsh environments, including situations with high/low temperatures,^[^ ^17^ ^]^ high salinity,^[^ ^18^ ^]^ high/low humidity,^[^ ^19^ ^]^ and ultrahigh pressures,^[^ ^20^ ^]^ are in increasing demand.^[^ ^21^ ^]^ For example, biometric data (e.g., heart rate, respiration rate, body temperature, and electrocardiogram) collected by physiological monitoring of firefighters on the fireground can provide near‐real‐time information for the commander to make critical decisions about the dangerous level of the members operating on the scene, which will enhance a firefighter's safety and survivability. However, current high‐temperature sensors based on silicon,^[^ ^22^ ^]^ diamond,^[^ ^23^ ^]^ and ceramics,^[^ ^24^ ^]^ are mechanically rigid and costly, while polymer‐based flexible sensors undergo severe performance degradation or even complete failures under complicated environments. Thus, conformable and reliable flexible pressure sensors that can operate under harsh conditions will greatly expand their application fields.

To achieve the above purpose, interconnected ceramic networks built with quasi‐1D structures, such as nanobelts and nanofibers, have been suggested as potential candidates for flexible ceramic sensors, offering the possibility of transforming bulky brittle ceramics into large‐area flexible thin films.^[^ ^25^, ^26^ ^]^ Such ceramic nanofibrous networks give a very high degree of porosity as well as extra lightweight, serving ideally for breathable and wearable devices. In comparison with polymers whose applications are exclusively confined to benign conditions, ceramics are inherently resistant to aggressive environments, including severe physical and corrosive chemical circumstances. Moreover, hysteresis with increased relaxation time that is caused by the viscoelastic creep of polymer chains can be significantly minimized in ceramic networks, which greatly enhances the response speed and performance stability of pressure sensors. In this work, titanium dioxide (TiO_2_) nanofibers are selected as the dielectric material for a sandwich‐structured capacitive pressure sensor owing to their high melting point and excellent mechanical properties. The pressure sensor exhibits good mechanical flexibility, high pressure‐sensitivity, ultralow limit of detection, and fast response speed. Different from previous studies where TiO_2_ serves as a functional semiconductor for gas and chemical sensing purposes,^[^ ^27^, ^28^ ^]^ the TiO_2_ nanofibers in this work are used as structural building blocks of the sensing architecture based on an electromechanical sensing mechanism. Thus, the capacitance‐to‐pressure sensitivity of the sensor studied here is determined by the compressive properties and the concomitant change of the effective dielectric constant, which are inert to humidity, light intensity, and chemical species. Such ceramic‐nanofibrous‐network‐based sensors outperform polymeric counterparts in both cyclic performance and harsh environmental stability, which are demonstrated with examples of wearable health monitoring, motion detection, and reliable pressure sensing performance at high temperatures up to 370 °C.

Large‐area flexible TiO_2_ nanofibrous networks were prepared by electrospinning as shown in **Figure** **1**a. The preparation details are described in the Experimental Section. By adjusting the parameters of precursor solution, electrospinning process and heat treatment, TiO_2_ nanofibrous networks with different thicknesses were obtained, denoted as TiO_2_‐10, TiO_2_‐15, TiO_2_‐20, and TiO_2_‐30, which correspond to the electrospinning time of 10, 15, 20, and 30 min with an average thickness of 25, 47, 83, and 114 µm, respectively (see Figures S1–S3, Supporting Information). The resultant ceramic networks are free‐standing and mechanically flexible, which are composed of highly entangled continuous nanofibers (Figure 1b,c). The TiO_2_ nanofibers are uniform with an average diameter of 120 nm. A transmission electron microscopy (TEM) image with corresponding selected area electron diffraction (SAED) pattern reveals the polycrystalline nature of the TiO_2_ nanofibers, showing nanosized crystallites with well‐resolved lattice fringes (Figure 1d; and Figure S4, Supporting Information). X‐ray diffraction (XRD) results further confirm the anatase structure of the TiO_2_ nanofibers (Figure 1e).

**Figure 1:**
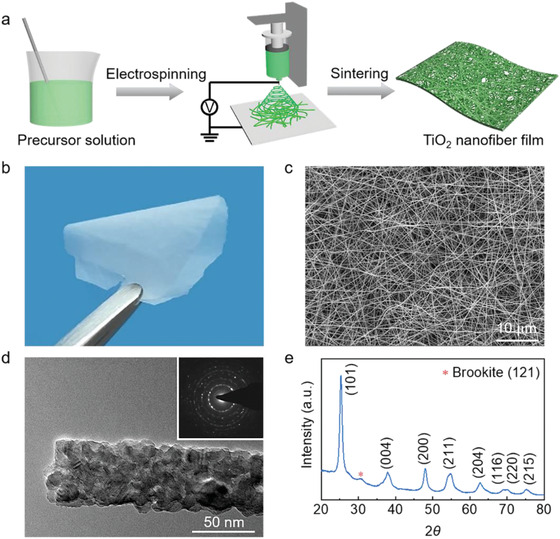
Fabrication and microstructure characterization of ceramic nanofibrous networks. a) The fabrication process of the TiO_2_ nanofibrous network. b) A photograph of an ultrathin TiO_2_ nanofibrous network. c) SEM image, d) TEM image (inset is the corresponding SAED pattern), and e) XRD spectrum of the TiO_2_ nanofibrous network.

By transferring the free‐standing TiO_2_ nanofibrous network in between a pair of flexible electrodes, i.e., polyimide (PI) films coated with silver nanowires (AgNWs), a simple sandwich‐structured capacitive pressure sensor was fabricated (**Figure** **2**a). For comparison, sensors based on poly(vinylidene fluoride) (PVDF) and poly(vinyl alcohol) (PVA) nanofibrous networks of the same thickness (25 µm) were prepared (Figure S5, Supporting Information). The capacitance‐to‐pressure sensitivity is defined as *S* = (Δ*C*/*C*_0_)/*P*, where Δ*C* is the capacitance change, *C*_0_ is the original capacitance, and *P* is the applied pressure. As shown in Figure 2b, the capacitance–pressure curve of the TiO_2_ nanofibrous network exhibits three distinct regimes. In the first regime, *P* < 0.3 kPa, capacitance has a sharp increase with increasing pressure, corresponding to the rapid decrease of the thickness of the network. After that, there is a slow‐down transition region from 0.3 to 13 kPa due to increased compression impedance. Finally, the network gets densified, marked with a slow rise of capacitance at *P* > 13 kPa. The three regimes have a pressure sensitivity of 4.4, 0.073, and 0.015 kPa^−1^, respectively, which are superior to that of using the PVDF and PVA networks. Such a distinction of sensitivity can be attributed to the larger dielectric constant of TiO_2_ and the smaller initial volume fraction of the nanofibers in the porous dielectric film (see the Supporting Information for theoretical derivations). Besides, owing to the low viscoelasticity of TiO_2_ nanofibers, low hysteresis was also observed in the capacitance–pressure loop of the TiO_2_ sensor (Figure S6, Supporting Information).

**Figure 2:**
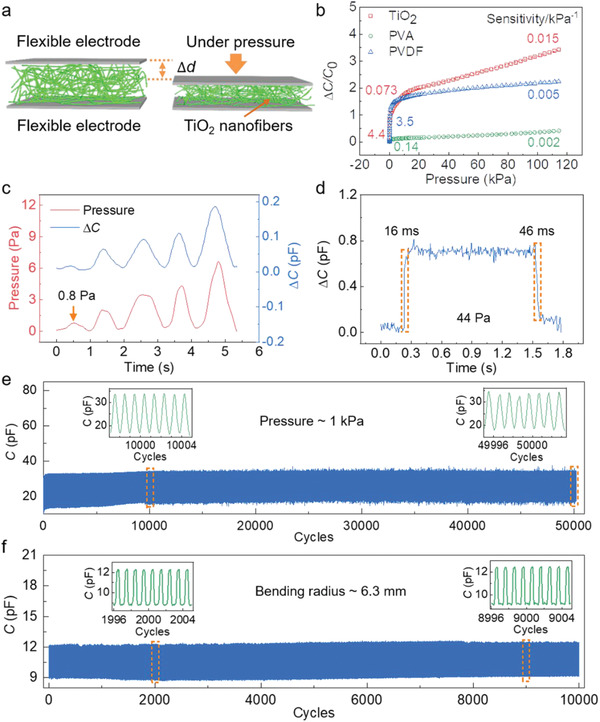
Pressure sensing properties of ceramic nanofibrous‐network‐based flexible sensors. a) Schematic of the flexible pressure sensor based on nanofibrous networks. b) Pressure sensitivity of sensors applying TiO_2_, PVA, and PVDF nanofibrous networks with the thickness of 25 µm. c) The limit of detection and d) the response/relaxation time of the TiO_2_‐10 sensor. e) Cyclic compression test of the TiO_2_‐10 sensor under a pressure of 1 kPa for 50 000 cycles. The insets show reversible responses at 10 000 and 50 000 cycles. f) Cyclic bending test of the TiO_2_‐10 sensor under a bending radius of 6.3 mm for 10 000 cycles. The insets show reversible responses at 2000 and 9000 cycles.

The pressure sensitivity can be further tuned by adjusting the thickness of the TiO_2_ nanofibrous network, exhibiting a higher *S* in the high‐pressure regime for thicker nanofibrous samples (Figure S7, Supporting Information). Moreover, as shown in Figure 2c,d, the TiO_2_ nanofibrous network sensor can detect tiny pressures down to <1 Pa and exhibits a fast response speed of 16 ms. The pressure sensing performance of the TiO_2_ nanofibrous network sensor surpasses the capabilities of biological skins in terms of sensitivity (0.078–0.018 kPa^−1^),^[^ ^29^ ^]^ limit of detection (2 kPa),^[^ ^30^ ^]^ as well as response speed (30–50 ms),^[^ ^31^ ^]^ showing great promise for electronic‐skin applications. Comparable sensing performances were also observed in TiO_2_‐15, TiO_2_‐20, and TiO_2_‐30 samples (see Figures S8 and S9 in the Supporting Information).

Remarkably, the TiO_2_ sensor exhibits high‐performance stability with a consistent response over 50 000 compressive and 10 000 bending cycles, highlighting the outstanding structural robustness of the ceramic nanofibrous network (Figure 2e,f). During the cyclic compression test, the original capacitance *C*_0_ drifts from 10.566 to 16.879 pF after the first 10 000 compressive cycles, and stays at 17–18 pF with a consistent signal capacitance *C*_p_ of ≈34 pF at 1 kPa for the next 40 000 cycles (Table S1, Supporting Information), suggesting that after an initial “training” process the sensor can reach a stable state for long‐term applications.

To provide further insight into the remarkable performance robustness of the TiO_2_ nanofibrous sensor, TiO_2_ along with PVDF and PVA nanofibrous networks of the same thickness were subjected to cyclic compression tests. As shown in **Figure** **3**, the TiO_2_ network exhibits a small plastic strain of 2.2% after 100 cycles at a compressive strain (*ε*) of 10%, while PVDF and PVA present higher residual strains of 7.0% and 8.4%, respectively. The maximum stress for the TiO_2_ nanofibrous network is 1.86 kPa at 10% strain, and 23.9 kPa at 60%, which are superior to those of PVDF (18.8 Pa at 10% and 667.1 Pa at 60%) and PVA polymer networks (7.9 Pa at 10% and 65.5 Pa at 60%, see Figure S10 in the Supporting Information). Besides, the TiO_2_ nanofibrous network maintains over 85% of its initial maximum stress after 100 compressive cycles in stark contrast with 48% for PVDF, and 20% for PVA. The large plastic deformations of the PVDF and PVA networks originate from the viscoelastic behavior of polymers, which may lead to a rapid performance degradation in the polymer pressure sensors. In comparison, the TiO_2_ nanofibrous network exhibits superior compressive properties due to the structural elasticity of ceramic nanofibers, which ensures a reliable performance for long‐term applications.

**Figure 3:**
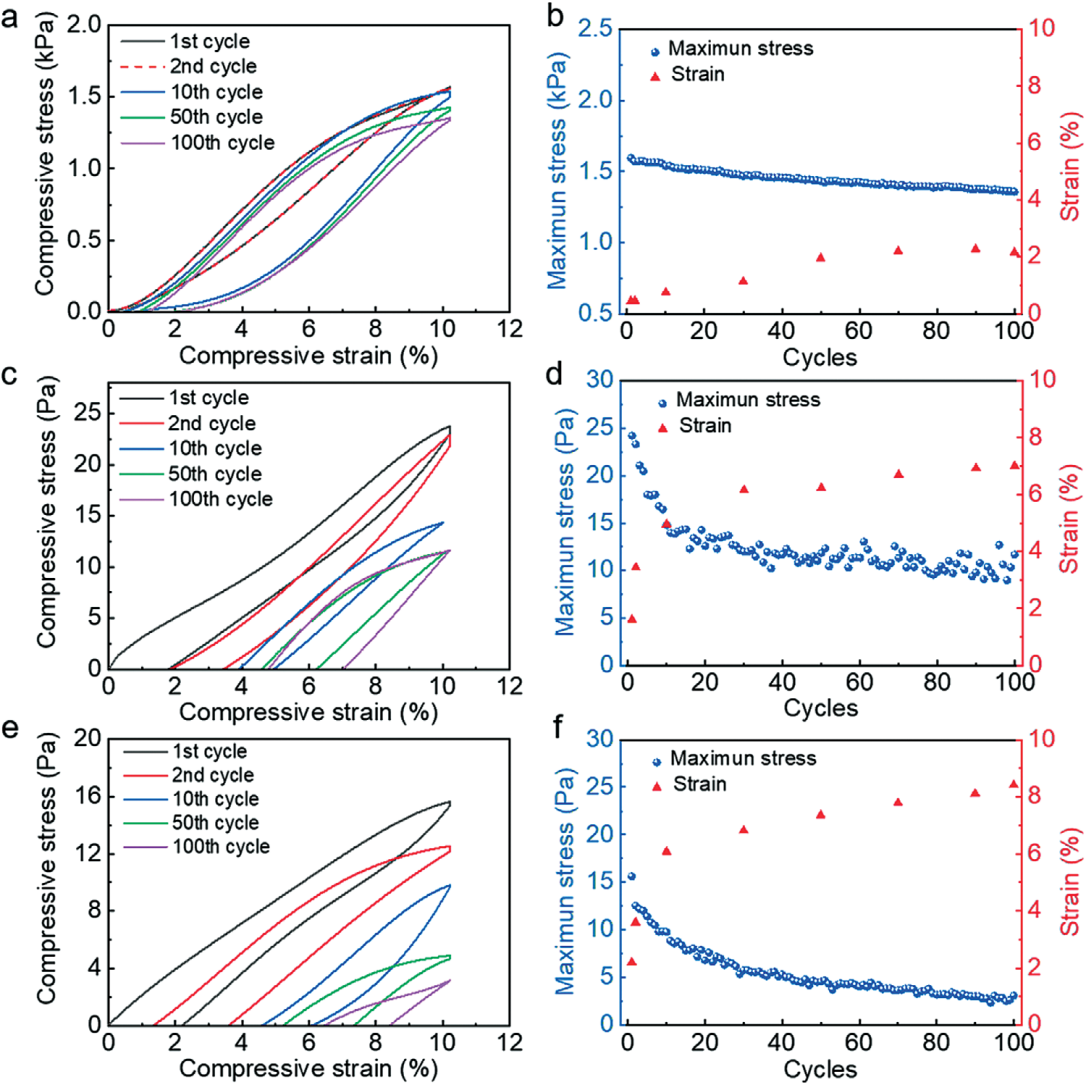
Compressive mechanical properties of the ceramic nanofibrous network versus polymer nanofibrous networks. a,c,e) Cyclic compressive stress–strain (*σ*–*ε*) curves of TiO_2_ (a), PVDF (c), and PVA (e) nanofibrous networks of the same thickness at the strain of 10%. b,d,f) Maximum stress and strain of TiO_2_ (b), PVDF (d), and PVA (f) nanofibrous networks at the strain of 10% under 100 compression cycles.

By employing conductive textiles as the electrodes, a fully breathable flexible pressure sensor was designed and constructed, as shown in **Figure** **4**a. The conductive textiles can be readily obtained with commercial fabrics by uniformly spraying a layer of AgNWs on one side (Figure 4b). Owing to the high porosity of the TiO_2_ nanofibrous network, the resultant capacitive sensor is highly breathable with excellent water‐vapor permeability. As shown in Figure 4c, the water evaporation rate of the breathable TiO_2_ sensor is comparable to the pure conductive fabric, giving a rapid decrease of the water content by 57.6% after being placed on a hot plate at 65 °C for 48 h, in stark contrast with the PI film (PI films are widely adopted as the substrate for flexible electronics) which shows only a slight decrease of 7.3%. Such lightweight, all‐network‐based, breathable pressure sensors are ideal for intimate skin‐attachable applications, which may avoid skin inflammation for continuous physiological monitoring.

**Figure 4:**
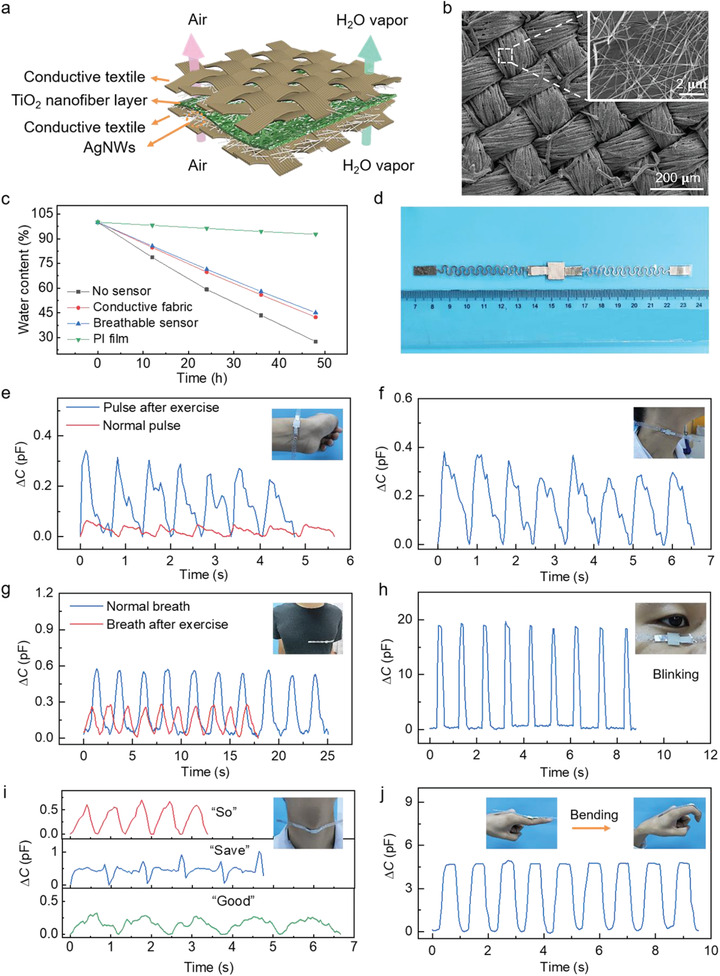
Breathable and wearable sensors for physiological monitoring and motion detection. a) Schematic of the breathable and wearable sensor. b) SEM images of conductive textiles coated with AgNWs at different magnifications. c) Water evaporation experiment of the breathable sensor. d) Photograph of the breathable and wearable pressure sensor (size of the sensing unit:10 × 10 mm^2^). e–j) Real‐time and in situ human physiology monitoring: radial pulse under normal and exercise conditions (e), the carotid pulse (f), respiration before and after exercise (g), eye blinking (h), vibration of vocal cords for pronunciation of the words “save,” “good,” and “so” (i), and finger bending (j). Insets: photographs of a wearable sensor attached to the wrist (e), skin at the pressure point above the carotid artery (f), chest (g), eyelid (h), neck (i), and knuckle of the index finger (j).

To demonstrate the potential applications in wearable health monitoring and motion tracking, the breathable pressure sensor (Figure 4d) was directly attached to different parts of the human body. As shown in Figure 4e, the radial pulse waveform with three distinct characteristic peaks, i.e., percussion, tidal, and diastolic waves,^[^ ^32^ ^]^ can be readily read out from the sensor attached on the wrist. After active exercise, part of the tidal wave disappears and the capacitance change increases, which is due to the enhanced vasodilation, ventricular‐vascular coupling, and dilated muscle arteries.^[^ ^33^ ^]^ Meanwhile, the pulse rate of the subject increases from 74 to 88 beats min^−1^. The carotid pulse data collected from the sensor adhered to the human neck shows a pulse rate of 73 beats min^−1^, which is in good agreement with the radial pulse (Figure 4f). Respiration can also be monitored by placing the sensor on the chest, offering valuable information for the prediagnosis of pulmonary diseases as well as the assessment of sleep apnea.^[^ ^34^ ^]^ Figure 4g shows the breathing signals recorded before and after exercise, indicating slower but deeper respiration under normal conditions with a frequency of 24 times min^−1^.

Wearable pressure sensors are critical for real‐time human motion detection and tracking systems, offering firsthand data for performance analysis and behavior prediction in a wide range of fields, such as sports, physical rehabilitation, and human–machine interaction. As shown in Figure 4h, regular eye blinking can be detected with stable and intense signals, suggesting potential applications in driver drowsiness detection and stress level analysis. Voice recognition was also demonstrated by attaching the sensor to the neck, exhibiting distinct repeatable characteristic wave patterns when the tester articulated different words, e.g., “so,” “save,” and “good” (Figure 4i). The capability of voice recognition is enabled by the high sensitivity of the sensor under subtle pressures, such as the vibration of vocal cords, which may find applications in remote multimedia control and vocal rehabilitation assistance. Natural joint motions of finger joints, wrists, ankles, and elbows produce large strains with active muscle activities. Owing to the lightweight and compliant structure, the pressure sensor can conform to the finger knuckle and track finger movements without restricting the degrees of freedom at the joints (Figure 4j).

Flexible pressure sensors that can operate under extreme conditions would greatly advance their applications in harsh environments. Owing to the inherent harsh‐environment resistance of ceramics, a high‐temperature‐resistant flexible pressure sensor based on TiO_2_ nanofibrous networks was proposed. As shown in **Figure** **5**a, the dielectric TiO_2_ nanofibrous network is sandwiched between a pair of carbon fiber cloth, which is adopted as the electrode for its high‐temperature stability (Figure S11, Supporting Information). Notably, the TiO_2_ sensor can survive the butane flame with a high temperature up to ≈1300 °C and exhibits a reasonable pressure sensitivity of 0.003–0.008 kPa^−1^ over the pressure range of 0–310 kPa after burning (Figure 5b,d). The TiO_2_ sensor was also tested at elevated temperatures. As shown in Figure 5c,d, the TiO_2_ sensor tested at 370 °C shows a low‐pressure (0–40 kPa) sensitivity of 0.028 kPa^−1^ and a high‐pressure (75–310 kPa) sensitivity of 0.005 kPa^−1^, which are comparable to its sensitivity at room temperature (30 °C). In our experiment, the dielectric TiO_2_ nanofibrous network was prepared by calcination at 450 °C, below which the sensor can preserve its compressive properties due to the negligible change in its microstructures, thus exhibiting a stable performance at elevated temperatures. The results of the multicycle compression tests show that the sensor gives reversible and consistent signals in response to varied applied pressures at room temperature, 370 °C, and after burning in the butane flame (Figure 5e,f; and Figure S12, Supporting Information), confirming its performance stability at elevated temperatures.

**Figure 5:**
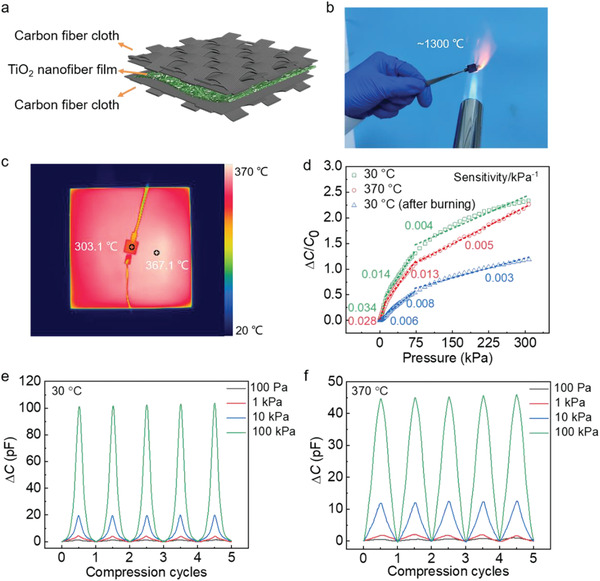
High‐temperature‐resistant pressure sensors. a) Schematic of the device structure of the high‐temperature‐resistant sensor. b) Photograph of the sensor burned in the butane flame (the temperature is up to ≈1300 °C). c) Thermal image of the sensor tested at 370 °C. d) Capacitance to pressure sensitivities of the sensor tested at 30, 370, and 30 °C after burning in the butane flame. e,f) Multicycle compressive tests with different peak pressures, showing reversible capacitance change at 30 °C (e) and 370 °C (f).

In summary, we have presented a highly sensitive and reliable flexible pressure sensor based on resilient ceramic nanofibrous networks through a scalable electrospinning process. Owing to the intrinsic elasticity of TiO_2_ nanofibers along with high structural connectivity, the ceramic sensors exhibit superior mechanical resilience with minor residual strains after cyclic compression, which accounts for the excellent performance stability in fatigue tests. Besides, the ceramic nanofibrous network demonstrates desirable properties, such as extra lightweight, excellent flexibility, high pressure‐sensitivity, and high liquid–vapor permeability, enabling continuous precise physiological monitoring by comfortable wearing without skin irritation. Moreover, the high‐temperature‐resistant flexible pressure sensor was demonstrated with the design of a full‐textile‐based sandwich structure composed of carbon fiber cloth electrodes and a ceramic nanofibrous dielectric layer, which can function properly under temperatures up to 370 °C and even survive the butane flame. The overall performance of the TiO_2_ nanofibrous sensor is superior to that of most breathable pressure sensors (Table S2, Supporting Information), which may advance the practical applications of wearable sensors for both daily activities and extreme conditions.

## Experimental Section

##### Preparation of TiO_2_, PVDF, and PVA Nanofibrous Networks and Flexible Pressure Sensors

For TiO_2_ nanofibrous networks, anhydrous ethanol and acetic acid with a mass ratio of 3:1, poly(vinylpyrrolidone) (*M*_w_ = 1 300 000) and tetrabutyl titanate (Ti(OBu)_4_) with a mass ratio of 1:1 were mixed and stirred for 4 h. Electrospinning was carried out at a high voltage of 16 kV and a pump speed of 15 µL min^−1^, and pristine nanofibers mat were collected from grounded aluminum foil, TiO_2_ nanofibrous networks were obtained after sintering at 450 °C for 2 h with a heating rate of 5 °C min^−1^. For PVDF nanofibrous networks, 15 wt% PVDF precursor solution was prepared by mixing 1.522 g PVDF (*M*_w_ = 530 000) with 6 mL acetone and 4 mL dimethylformamide (DMF), and then vigorously stirred in a water bath at 70 °C for 12 h. A high voltage of 18 kV and a pump speed of 30 µL min^−1^ were applied to electrospinning. PVDF nanofibrous networks were collected after 10 min of electrospinning. For PVA nanofibrous networks, 0.7 g (7 wt%) poly(vinyl alcohol) (*M*_w_ = 205 000) was dissolved in 9.3 g deionized (DI) water and the mixed solution was stirred in a water bath at 90 °C for 5 h. With a high voltage of 20 kV and a pump speed of 4 µL min^−1^, electrospun PVA nanofibrous networks were obtained after 15 min. In the above preparation process, the outer diameter of the syringe needle was 0.83 mm, the distance between the collecting plate and the tip of the syringe needle was 19 cm, and the relative humidity was 40%± 5%. TiO_2_, PVDF, and PVA nanofibrous networks with an average film thickness of 25 µm were adopted as the dielectric layer. PI films were cut by a laser cutting machine (WE6040, Waner Co. LTD) into round shapes with a diameter of 1.4 cm and then spray‐coated with commercial AgNWs (XFJ81, XFNANO) by a sliver jet compressor (IS‐50, Iwata). Silver wires were used as conductive lines and bonded to the electrodes by silver glue (SPI) and the sandwich‐structured pressure sensors were sealed with the 3M insulating tape eventually.

##### Mechanical Properties of TiO_2_, PVDF, and PVA Nanofibrous Networks

Compression tests were performed with a microcontrolled pressure testing system equipped with a 20 N load cell. The tested networks were cut into squares of a size of 7 × 7 mm^2^, which was also the size of the indenter. The *σ*–*ε* curves with *ε* of 10%, 20%, 30%, 40%, 50%, and 60% were tested at a strain rate of 0.05 mm min^−1^. The cyclic compression tests were performed at *ε* = 10% with a strain rate of 0.05 mm min^−1^ and a prestress of 1 mN.

##### Assembly and Characterization of Breathable and Wearable Pressure Sensors

One side of the as‐purchased cotton cloth was sprayed with AgNWs and then cut by a laser cutting system into squares of a size of 1 × 1 cm^2^. The wavy‐shaped aluminum foil with a thickness of 0.08 mm was used as the conductive lines, which was bonded to conductive fabrics by silver glue. The conductive textile electrodes with an average sheet resistance of 90.6 Ω sq^−1^ were obtained. Two conductive textile electrodes were bonded together with the 3M double‐sided tape adhered to the inner edge, and the TiO_2_‐30 with a size of 5 × 5 mm^2^ was placed in between the electrodes. The vapor permeability of the sensor was measured by covering a water‐contained bottle with sensors and placing it in the fume hood at 65 °C for 48 h. The results were compared with control samples including open bottles, conductive textiles, and PI films.

##### Fabrication of High‐Temperature‐Resistant Pressure Sensors

The carbon fiber cloth (W1S1009, CeTech Co., LTD) with a sheet resistance of 10.3 mΩ sq^−1^ was cut into squares by 1 × 1 cm^2^. The TiO_2_ nanofibrous networks with a size of 0.7 × 0.7 cm^2^ were placed in between the electrodes, which were bonded with high‐temperature glue (1853, Ausbond). The pressure sensitivity was tested at 30 °C, and 370 °C by placing the sensor on a hot plate that was positioned above the platform of the loading bar of the force gauge, and the electrodes of the sensor were connected to the LCR meter by a 1 m long heat‐proof test lead using an alligator clip. The temperature of the hot plate and the sensor was monitored with an infrared camera (220s, FOTRIC). A butane torch was used to offer the high‐temperature flame with a temperature up to ≈1300 °C.

##### Measurement

Capacitance measurements were taken at a frequency of 1 MHz with a 1 V AC signal, using a Keysight E4980AL Precision LCR Meter. For compressive tests, force and frequency were controlled by an automatic z‐axis stage loading bar with a force gauge (XLD‐20E, Guangzhou Precision Control Test Instrument Co. LTD) with a resolution of 0.00001 N at a compression rate of 2 mm min^−1^ and the pressure of 1 kPa for cyclic tests. A broad range of force (0.0001 N‐17.5 N) was programmed to measure the pressure sensitivity with a minimum force down to ≈0.0001 N for the measurement of the detection limits. Objects with a weight of 1.0 g (≈44 Pa) were employed to test the response/relaxation time. For bending tests, the force was controlled by a customized automatic x‐axis loading bar with a bending rate of 2 mm s^−1^ and a bending radius of 6.3 mm.

## Conflict of Interest

The authors declare no conflict of interest.

## Supporting information

Supporting Information
