# Supplementary material for: A Highly Sensitive, Reliable, and High‐Temperature‐Resistant Flexible Pressure Sensor Based on Ceramic Nanofibers
Source: Adv Sci (Weinh). 2020 Jul 27;7(17):2000258. doi: 10.1002/advs.202000258 (PMC7507114; doi:10.1002/advs.202000258)
Supplement: Supplementary file 1 — Supporting Information [file ADVS-7-2000258-s001.pdf]

Copyright WILEY-VCH Verlag GmbH & Co. KGaA, 69469 Weinheim, Germany, 2020.

Supporting Information

for *Adv. Sci.*, DOI: 10.1002/advs.202000258

A Highly Sensitive, Reliable, and High-Temperature-Resistant Flexible Pressure Sensor Based on Ceramic Nanofibers

Min Fu, Jianming Zhang, Yuming Jin, Yue Zhao, Siya Huang, and Chuan Fei Guo**

1. Mathematical Derivations for the Mechanical-to-Capacitive Sensitivity

In our case, the pressure-to-capacitive sensing model can be derived from the compression behavior of fibrous assemblies.[1] The relationship describing the compression behavior of the fibrous mass is

$$P = \lambda\left(\frac{1}{v^3} - \frac{1}{v_0^3}\right),$$ [1]

where v is the volume of the assembly, and v_0 is the value of v when P = 0. $\lambda = KEv_{\mathrm{f}}^3$ is a constant of proportionality with K represents fiber spatial distribution and characteristics. E is elastic modulus, and v_{f} is the volume of fibers. Since the relationship between the fiber volume fraction and volume of fibers is $V_{\mathrm{f}} = v_{\mathrm{f}}/v$, the relationship can be expressed as

$$P = KE\left(V_{\mathrm{f}}^3 - V_{\mathrm{f0}}^3\right)$$

where V_{f0} is the fiber volume fraction when P = 0.

By substituting V_{f} with the thickness of the fibrous dielectric layer (t) using $V_{\mathrm{f}} = V_{\mathrm{f0}} \cdot t_0/t$, the equation can be described as:

$$P = KEV_{\mathrm{f0}}^3\left[\left(\frac{t_0}{t}\right)^3 - 1\right]$$

where t_0 is the thickness of the fibrous dielectric layer when P = 0.

In this work, the pressure sensor is designed as a parallel plate capacitor with a composite dielectric layer consisting of nanofibers and air. Thus, the total capacitance C can be depicted as

$$C = (\varepsilon_{\mathrm{air}} V_{\mathrm{air}} + \varepsilon_{\mathrm{f}} V_{\mathrm{f}})\varepsilon_0 (A/t)$$

where V_{air} and V_{f} refer to the volume fraction of the air and nanofibers in the composite dielectric layer, $\varepsilon_{\mathrm{air}}$ ($\varepsilon_{\mathrm{air}} \approx 1$) and ε_{f} are the dielectric constant of air and nanofibers, respectively,

ε_0 is the permittivity of space, A is the overlapping area of the parallel electrodes. Given V_{air} = $1-V_f$, the capacitance of the sensor can be derived as:

$$C = \frac{\varepsilon_0 A}{t_0}\left[\left(\frac{P}{KEV_{f0}^3}+1\right)^{\frac{1}{3}} + V_{f0}(\varepsilon_f - 1)\left(\frac{P}{KEV_{f0}^3}+1\right)^{\frac{2}{3}}\right]$$

Thus, the capacitance is proportional to the dielectric constant of nanofibers, and the initial volume fraction of the nanofibers also has a great influence on the device sensitivity, i.e., nanofibrous networks with a large dielectric constant and a small initial fiber volume fraction would result in a high pressure sensitivity. TiO_2 has a dielectric constant ten times larger than PVDF and PVA. Besides, owing to the calcination process with concurrent large shrinkage of the precursor $Ti(OBu)_4$/PVP nanofibers, the resultant TiO_2 nanofibers are of a much smaller diameter (~120 nm), which leads to nanofibrous networks with a much higher porosity (i.e., low V_{f0}) than that of PVDF (~360 nm) and PVA (~220 nm) nanofibrous networks. Therefore, the TiO_2 nanofibrous sensor shows better performance in pressure sensitivity than the PVDF and PVA counterparts of the same film thickness.

2. Supplementary Figures and Tables

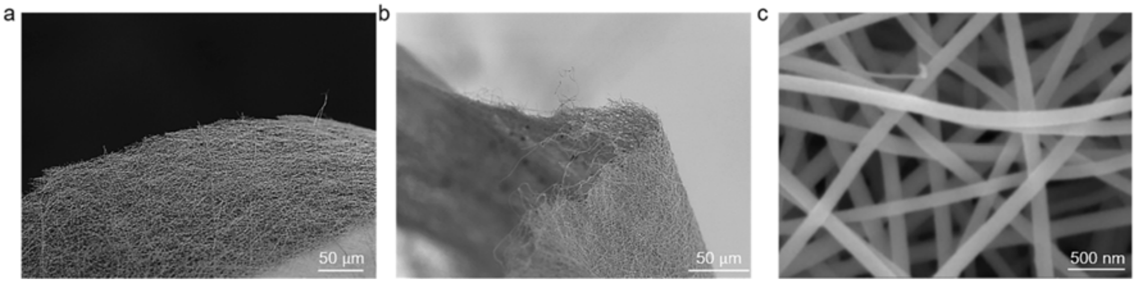

Figure S1. SEM images of ultrathin TiO_2 nanofibrous networks at different magnifications.

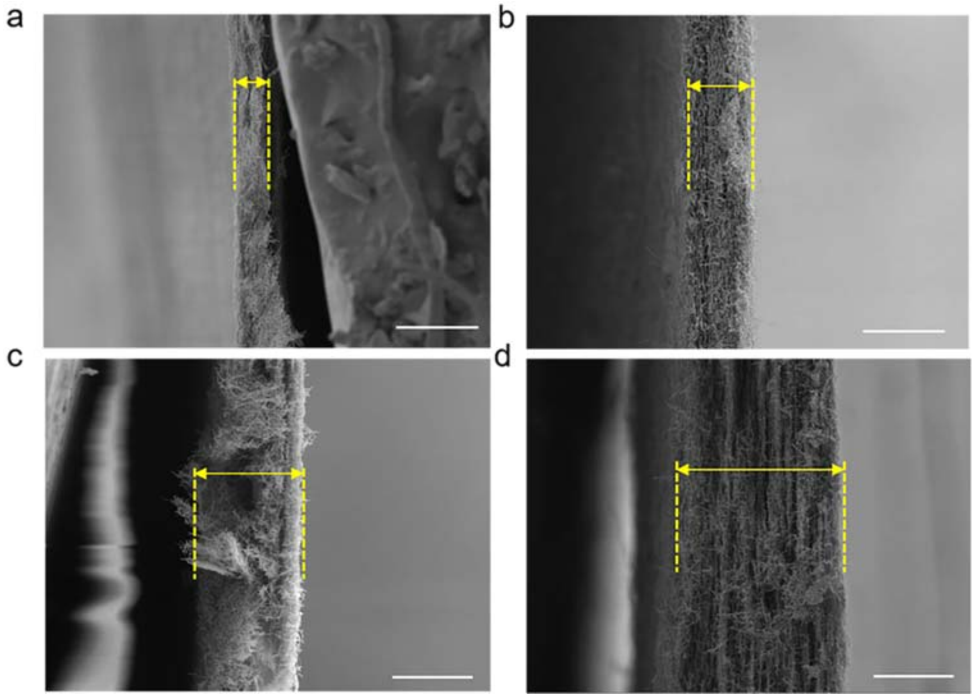

Figure S2. SEM images of the sectional view of TiO_2 nanofibrous networks by electrospinning for a) 10 min, b) 15 min, c) 20 min, and d) 30 min. Scale bars, 50 µm.

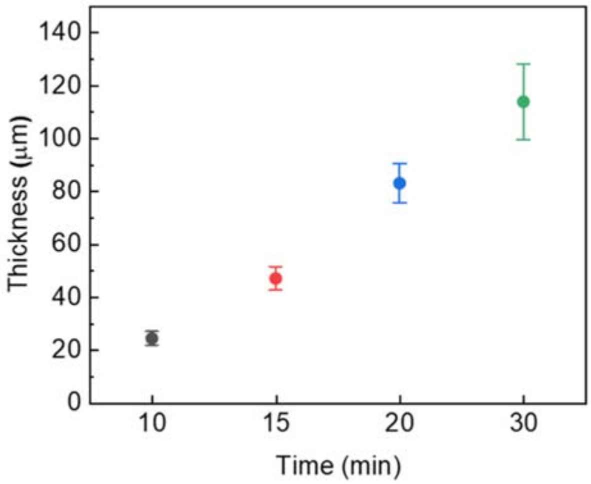

Figure S3. Thickness range of TiO_2 nanofibrous networks by electrospinning for 10, 15, 20, and 30 min.

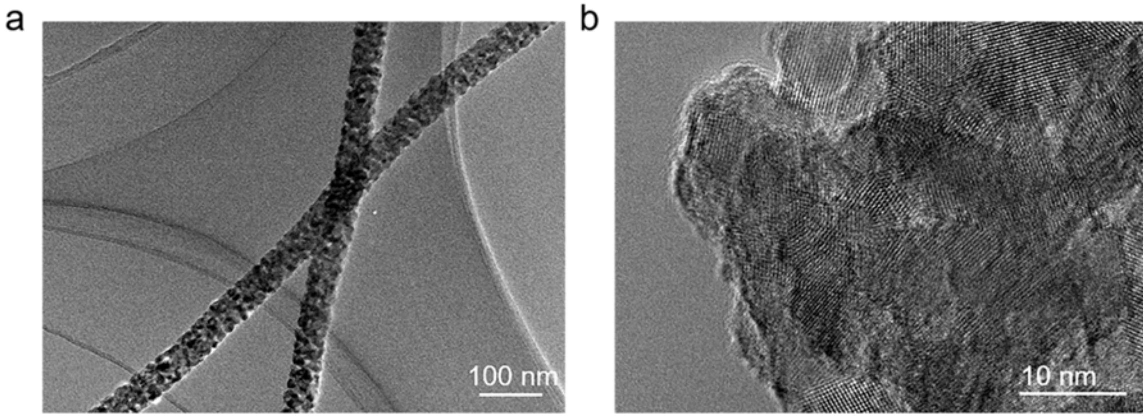

Figure S4. High-resolution TEM images of the TiO_2 nanofiber.

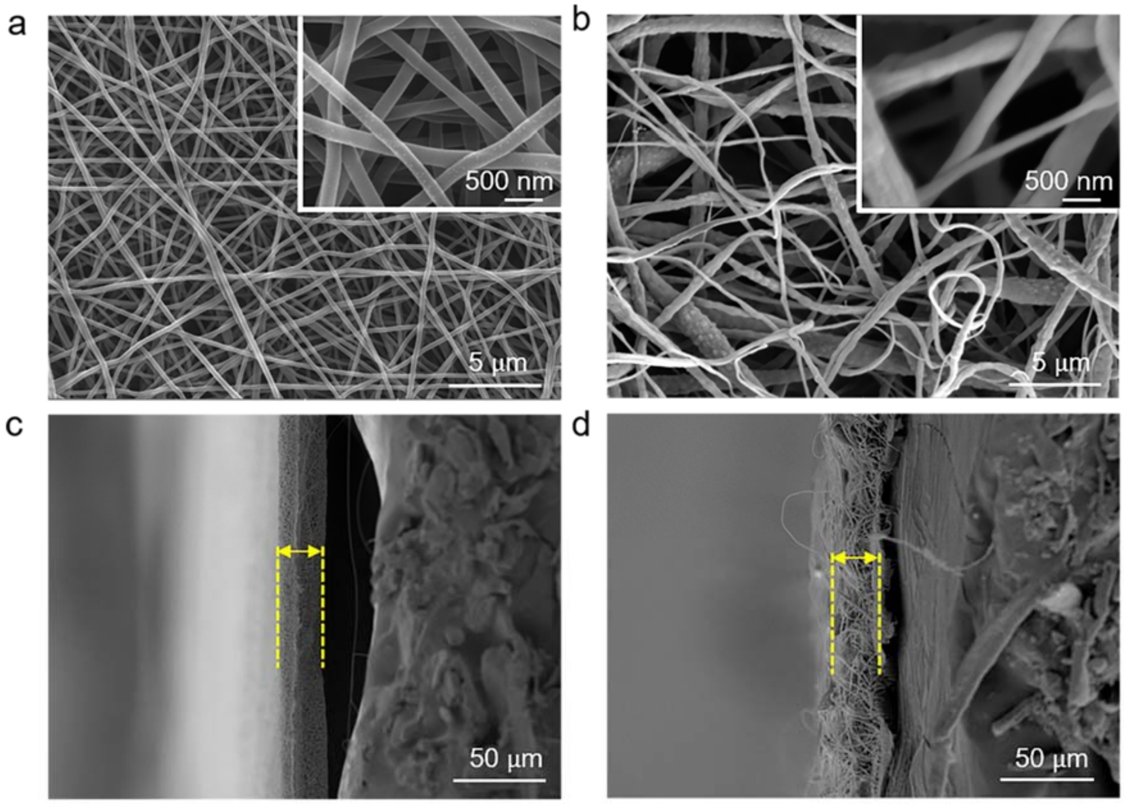

Figure S5. SEM images of a) PVA and b) PVDF nanofibrous networks. The corresponding cross-sectional SEM view of c) PVA and d) PVDF nanofibrous networks.

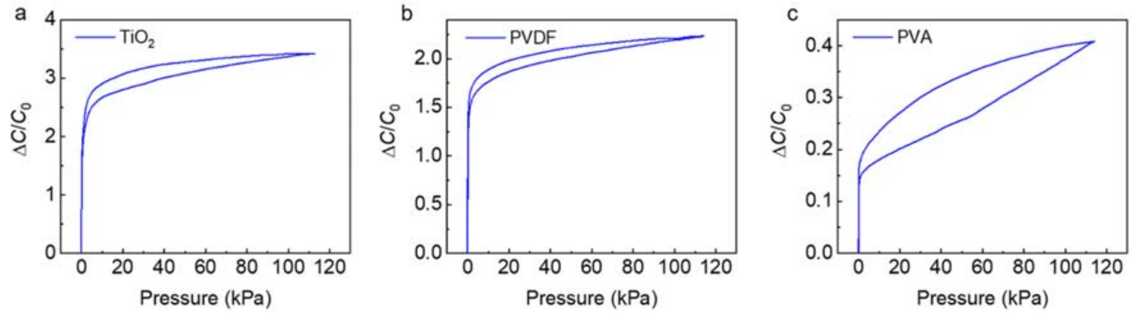

Figure S6. Pressure sensitivity of a) TiO_2, b) PVDF, and c) PVA nanofibrous sensors with the film thickness of 25 μm.

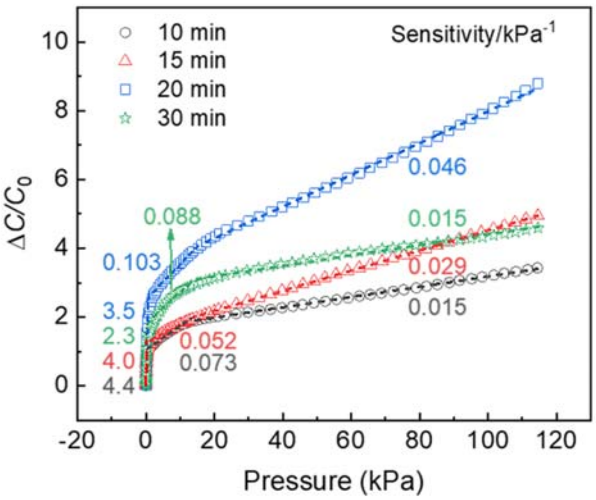

Figure S7. Pressure sensitivity of sensors with TiO_2 nanofibrous networks by electrospinning for 10, 15, 20, and 30 min.

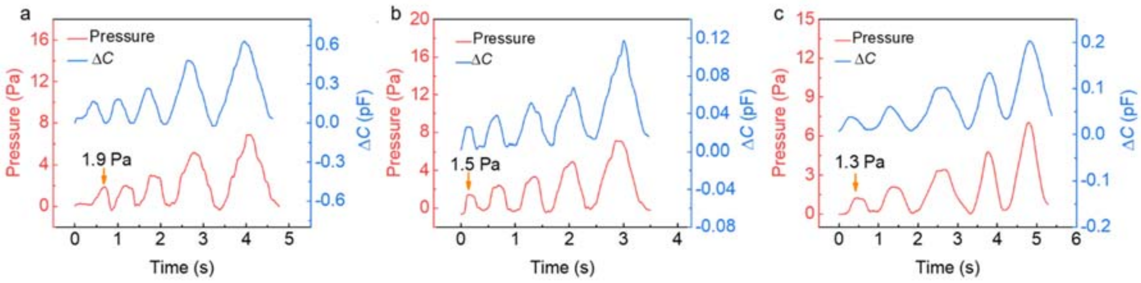

Figure S8. Detection limit of sensors with TiO_2 nanofibrous networks by electrospinning for a) 15 min, b) 20 min, and c) 30 min.

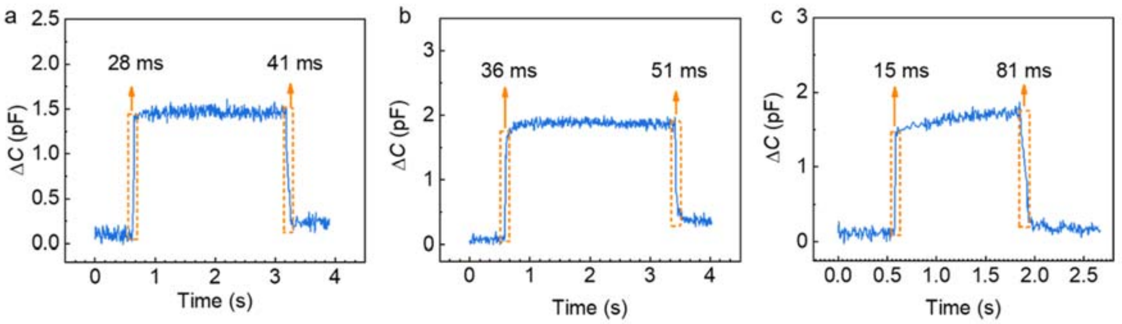

Figure S9. Response/relaxation time of sensors with TiO_2 nanofibrous networks by electrospinning for a) 15 min, b) 20 min, and c) 30 min.

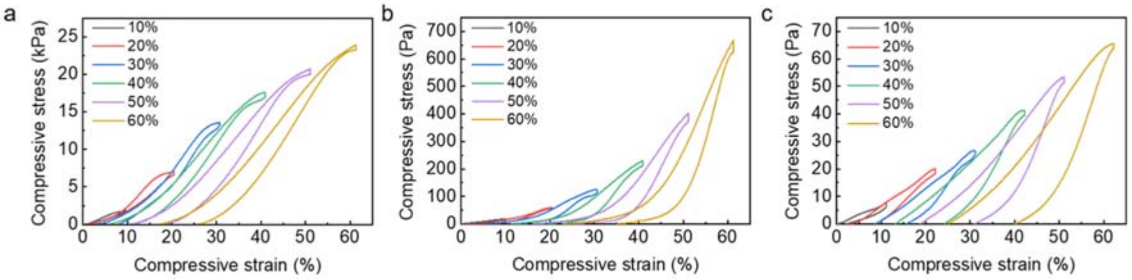

Figure S10. The stress-strain curves of a) TiO_2, b) PVDF, and c) PVA nanofibrous networks at strains of 10%, 20%, 30%, 40%, 50%, and 60%.

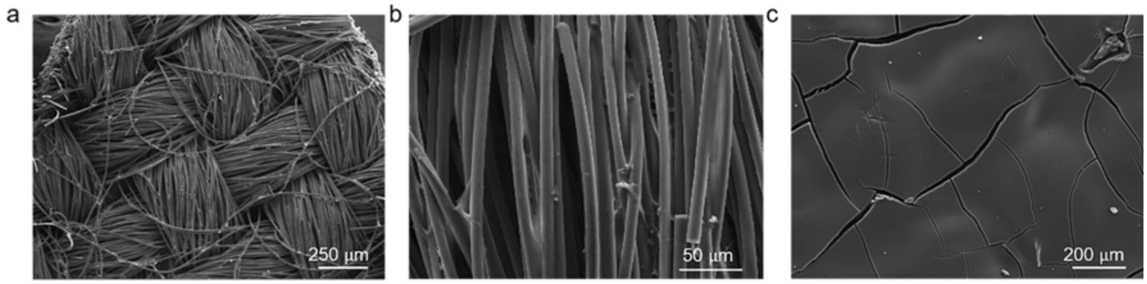

Figure S11. SEM images of a, b) front and c) back of the hydrophobic carbon fiber cloth.

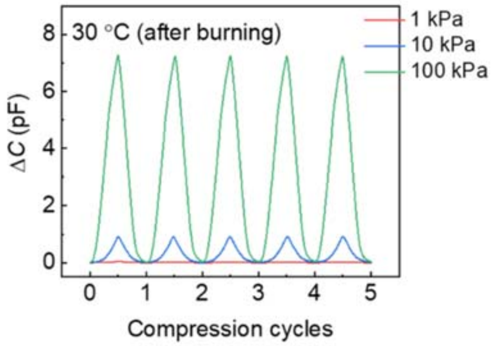

Figure S12. Multi-cycle compression tests of the capacitance change with different peak pressures at 30 □ after burning in the butane flame.

Table S1. C_0 and C_p at the pressure of 1 kPa for different compressive cycles.

Number of cycles	C_0/pF	C_p/pF
1	10.566	31.855
10000	16.879	33.726
30000	16.9	34.295
40000	18.256	34.298
50000	18.489	34.593

Table S2. The performance comparison between this work and previous studies on breathable pressure sensors.

Breathability	Sensitivity	Limit of detection	Response speed (ms)	Working temperature (□)	Cyclic stability (cycles)	Ref.
0.173 s mL^{-1}	4.2 kPa^{-1}	1.6 Pa	<26	RT	7 000	[2]
-	14.4 kPa^{-1}	2.0 Pa	24	RT	1 000	[3]
-	$<10^{-3}$ kPa^{-1}	tens of kPa	500	RT	250 000	[4]
6.16 mm s^{-1}	0.385 kPa^{-1}	2 600 Pa	-	RT	10 000	[5]
-	5.65×10^{6} kPa^{-1}	0.76 Pa	6	RT	1 000	[6]
3×10^{8} mL m^{2} for 24 h	474.8 (gauge factor)	-	33	RT	5 000	[7]
50.1 mm s^{-1}	-	0.06 N	-	RT	10 000	[8]
-	0.31 kPa^{-1}	200 Pa	20	RT	10 000	[9]
-	0.49 kPa^{-1}	20 Pa	30	RT	600	[10]
2×10^{4} mL m^{2} for 24 h	4.4 kPa^{-1}	0.8 Pa	16	RT-370	>50 000	This work

Reference

[1] J. Hu, *Structure and Mechanics of Woven Fabrics*, CRC Press, Cambridge, Massachusetts, USA **2004**.

[2] W. Yang, N.-W. Li, S. Zhao, Z. Yuan, J. Wang, X. Du, B. Wang, R. Cao, X. Li, W. Xu, Z. L. Wang, C. Li, *Adv. Mater. Technol.* **2018**, *3*, 1700241.

[3] M. Liu, X. Pu, C. Jiang, T. Liu, X. Huang, L. Chen, C. Du, J. Sun, W. Hu, Z. L. Wang, *Adv. Mater.* **2017**, *29*, 1703700.

[4] S. Honda, Q. Zhu, S. Satoh, T. Arie, S. Akita, K. Takei, *Adv. Funct. Mater.* **2019**, *29*, 1807957.

[5] R. Cao, J. Wang, S. Zhao, W. Yang, Z. Yuan, Y. Yin, X. Du, N.-W. Li, X. Zhang, X. Li, Z. L. Wang, C. Li, *Nano Res.* **2018**, *11*, 3771.

[6] Z. Zhou, Y. Li, J. Cheng, S. Chen, R. Hu, X. Yan, X. Liao, C. Xu, J. Yu, L. Li, *J. Mater. Chem. C* **2018**, *6*, 13120.

[7] Z. Song, W. Li, Y. Bao, F. Han, L. Gao, J. Xu, Y. Ma, D. Han, L. Niu, *ACS Appl. Mater. Interfaces* **2018**, *10*, 42826.

[8] H. Li, W. Zhang, Q. Ding, X. Jin, Q. Ke, Z. Li, D. Wang, C. Huang, *ACS Appl. Mater. Interfaces* **2019**, *11*, 38023.

[9] K. Kim, M. Jung, S. Jeon, J. Bae, *Smart Mater. Struct.* **2019**, *28*, 065019.

[10] Z. Wang, Y. Si, C. Zhao, D. Yu, W. Wang, G. Sun, *ACS Appl. Mater. Interfaces* **2019**, *11*, 27200.
